# Cases in Surgery, with Clinical Remarks Delivered at the Philadelphia Hospital

**Published:** 1861-07

**Authors:** S. D. Gross

**Affiliations:** One of the Surgeons to that Institution


ART. II.—*Cases in Surgery, with Clinical Remarks delivered at the Philadelphia Hospital.* By S. D. GROSS, M.D., one of the Surgeons to that Institution.

Reported by MR. THOMAS C. BRAINERD.

*Stricture of the Urethra cured by Internal Division.*

*Case* 1.—Michael McS., aged sixty-four, a farm laborer, of irregular habits, had suffered for fifteen years from difficulty in passing his water. For some months this had been upon the increase, and the stream had rapidly diminished in size, until it could be maintained only by the strongest effort. He experienced at all times a dull pain over the region of the bladder, and every attempt to void his urine produced also a distressing burning sensation in the perineum. He was obliged to pass water about ten times in the twenty-four hours, rising for that purpose frequently through the night. His general health was somewhat impaired, and he had great depression of spirits.

On examination, a firm, unyielding stricture was discovered in the body of the penis, about four inches behind the anterior orifice, which resisted the passage even of the smallest bougie. The patient had had gonorrhœa thirty years previously, from which he recovered with difficulty, but he knew of no immediate cause for the affection at the time of its appearance, and his stout, muscular frame seemed to indicate an excellent natural constitution.

*Case* 2.—The second patient, Robert W., was a tall, well-made man, a tailor by trade, thirty-six years old, who contracted gonorrhœa fifteen months since, but made such a rapid recovery that he thought no more about it. But four months afterward he noticed that the scalding reappeared in micturition, which soon became laborious and painful, as well as more frequent. His urine, at the time of his admission to the hospital, came away in drops, so that he occupied nearly half an hour in emptying his bladder, yet he was obliged to do this three or four times every night and about twice as often during the day.

His stricture was situated in the membranous portion of the urethra, just behind the bulb, was perfectly impassable to any instrument, and from the manner in which it held the point of the bougie conveyed the impression of great firmness.

*Case* 3.—Charles S., about twenty-seven years of age, a slender, feeble man, of sedentary habits, also suffered with gonorrhœa three years ago, and had never been able since to urinate with any comfort. The stream had steadily grown smaller, until it was now no larger than a small straw, while the patient was constantly tormented through the day by uneasy sensations, and occasionally experienced sharp twinges of pain. By straining he had brought on some prolapse of the rectum, which was increased by the costive state of his bowels. He was considerably emaciated, and felt incapacitated for any active exertion. The stricture was located a little anterior to that of the last patient, at the termination of the bulb, and, like both the others, was hard and unyielding. The urine at times was tinged with blood, and the urethra throughout its whole course was so sensitive that the scalding was an intense annoyance.

*Operation.*—A precisely similar operation was employed in each of these three cases. The patient, having all the clothing removed from his lower limbs, was placed upon his back, with the shoulders and pelvis slightly elevated, the thighs flexed upon the body, and the legs upon the thighs, the knees being widely separated. Chloroform was then administered, and a thorough examination made of the situation, firmness, and, as far as could be ascertained, the extent of the stricture; using for this purpose a silver catheter, having a somewhat conical point, as being lighter than a metallic bougie, yet sufficiently strong and inflexible.

The urethrotome, about the diameter of a No. 10 catheter, but somewhat less curved, had the bulb containing the concealed blade half an inch behind the point, to which it diminished gradually. The cutting edge was on the upper or concave side of the instrument, although Professor Gross generally prefers it upon the lower; the stilet retracted the blade into its sheath as soon as the pressure was removed from the knob at its other extremity. The point of the canula being carefully advanced as far as possible into the constricted portion of the urethra, the knife was projected, great care being taken at the same time to preserve the handle of the instrument in the median line of the body, to diminish the danger of making a false passage. The stilet was then allowed to spring back, and the extremity again pressed forward, repeating the incision if necessary, and slowly continuing this process until the loss of resistance gave evidence that the stricture was completely divided. In the first and last cases one incision only was required; in the second, however, both the base and sides of the urethra were notched several times before the obstacle was overcome. As soon as it was passed, the urethrotome was withdrawn and a catheter of rather larger diameter inserted, which slipped at once without difficulty into the bladder.

*After-treatment and results.*—The retention of the catheter being provided for, by securing it to a double T-bandage, a full anodyne was directed in case of pain, and the patient confined to bed. The following day the instrument was taken out, cleaned, and reinserted, the process being repeated daily until the urethra regained its natural character.

No fever or ill consequences of any sort appeared in any of the cases, but, on the contrary, the health of each patient began to improve almost from the moment of the operation. Three weeks afterward, when they were again brought before the class, each of them said that he had not suffered either pain or inconvenience, and that what had been before the greatest agony had become a pleasure from the ease and force with which he was able to project a stream of almost fabulous dimensions.

*Remarks.*—In connection with these cases, Professor Gross offered the following observations:—

The partial diminution of the calibre of the urethra, to which we give the name of stricture, is either temporary and paroxysmal, caused by spasm, or the result of an effusion of lymph, upon, in, or under the lining membrane, in which case it is termed organic, and is permanent. Only the latter requires surgical treatment, which must be modified to meet the age of the stricture, which is soft and elastic when recent, but hard and unyielding when time has effected its complete organization. Ordinarily its form resembles the narrowing produced by a ligature cast around a membranous tube; but not unfrequently it is more or less accumunated on one side, giving a zigzag course to the canal, or even stretching across like a bridge, making two distinct passages. In extent also there is great diversity; the constricted portion being sometimes not over a line in length, at others several inches, while it is not uncommon for the tube to be affected simultaneously in several different places.

This plastic effusion may arise from a traumatic cause, but most frequently it is the result of long-continued inflammation of the urethra; either simple, or, as is the fact in ninety-nine cases out of a hundred, produced as an immediate effect of gonorrhœa, or of its unskillful treatment. There is, however, a bad variety of stricture, very apt to follow chancre, situated at the anterior urinary orifice, in which the narrowing is close behind it. Otherwise it may be located in any part of the tube, but is most frequently found near the junction of the bulbous and membranous portions.

The direct consequences of this affection are painful and difficult urination, with a greater frequency in its necessity. But the violent and oft-repeated straining soon produces hypertrophy of the muscular and mucous coats of the bladder, and a sacculated or columnar condition of that organ, with an inflammation which, not unfrequently extending along the ureters to the kidneys, produces enlargement, suppuration, and an accumulation of pus in those organs, followed eventually by death. The urethra behind the stricture also becomes dilated, abscesses often forming which, breaking externally, lead to perineal fistules; occasionally, also, the prostate gland, seminal vesicles, and even the testes participate in the inflammatory action. In old cases the rectum is often relaxed so as to prolapse, or become the seat of hemorrhoidal tumors. These accumulated annoyances, with the pain which necessarily attends the affection, soon break down the strongest constitution, and life is rendered miserable, if it is not destroyed.

Characteristic as the difficulty and frequency of micturition and the diminished stream may be, it is yet impossible to form a correct diagnosis of stricture without making an examination as to the patency of the canal by means of some instrument. The best for this purpose is the ordinary silver catheter, the graduated staff of the French surgeons being entirely unnecessary, since, if it is required to ascertain the exact depth of the constriction, nothing is better than to mark it on a wax bougie with the finger or thumb nail.

In strictures which are comparatively of short standing, the ordinary process of dilatation is sufficient to produce a cure; but if the obstruction has remained long enough to be thoroughly organized, there is nothing so efficacious in securing a rapid and permanent deliverance as its division. The metallic bougies usually employed in the former case are much too heavy, nothing answering the purpose so well as a silver catheter, choosing one of small size at first, which should be very gently pushed down to the seat of the affection, through the stricture, and on into the bladder. This instrument has also the great advantage of being permeable, so that it can remain in the canal, if necessary, without incommoding the patient. On the second day the same instrument should be reinserted, and kept in the passage for some minutes, or even an hour, and then one of larger size substituted and almost immediately withdrawn; continuing this process day by day, gradually increasing the size of the catheters until the urethra acquires its natural dimensions. Or, as this generally occupies several months, the several sizes of catheter may be used in rapid succession at one operation; but, although this method succeeds admirably with proper care, it is apt to excite violent inflammation of the urethra, requiring the most active measures before it can be subdued. If the stricture is near the external orifice, and very soft, excellent results are often obtained from using a bougie of slippery elm, which, being allowed to remain in the passage, becomes moist and swollen, thus rapidly accomplishing the necessary dilatation. But whatever method be employed, there is an invariable necessity of introducing, at regular though increasing intervals after recovery, a large-sized instrument into the bladder, as otherwise there will inevitably be a reproduction of the disease.

Far preferable in the case of old, hard strictures is the operation by division; since this does not act mechanically only, for the knife is the most powerful alterant within reach of the surgeon, and such is the local impression made by it, that the absorbents are immediately stimulated to act upon the abnormal mass of effused lymph, and the consequence is a cure, at once permanent and certain.

In the three cases before us, it would take an experienced operator perhaps months to accomplish by dilatation what was here done in a few minutes, much more efficaciously, for the chances of recurrence are greatly diminished. All the constitutional depression arising from long protracted treatment is avoided, and the patient is at once transported from a state of misery to one of comfort. The chief danger of the operation is the risk of making a false passage, but this is easily avoided by delicate manipulation, guided by a thorough knowledge of the anatomy of the parts. The internal incision is readily effected in any part of the canal by the use of the urethrotome, or, if the stricture is near the orifice, by a narrow, straight, blunt-pointed bistoury, and is much less liable to be followed by infiltration and other bad consequences, than division from the exterior. In very many cases the constriction is so tight or the passage so tortuous, that it is impossible to introduce even the delicate staff of Professor Syme, and unless the stricture is completely impervious, easily distinguished from the outside, or complicated with fistulous sinuses, perineal section is generally unnecessary. Such, however, is the delicacy of this part of the human organism that, whatever mode be employed, the practitioner must keep a close watch on the ensuing phenomena, and be prepared to meet at once and energetically the first unfavorable symptom.

*Sloughing Chancre.*

Michael A., aged twenty-eight, a married man, of muscular frame and good constitution, by trade a harness-maker, entered the Hospital on the eighteenth of February. According to his own story, he had impure connection three weeks previously, followed by a sore just behind the corona of the head of the penis, which so troubled his mind that he commenced drinking, and was in consequence unable to give any further history of his disease. He only remembered that in his more sober moments, he felt intense pain, and that there had been a very abundant discharge of pus.

When admitted, the whole prepuce was hanging as a dead and blackened mass below the penis, the head of that organ was greatly enlarged and inflamed, and an angry, sloughing ulcer covered more than half of the remaining portion. It was bathed in an abundant, thin discharge, was the seat of constant pain, and, except a few granulations, showed no evidences of any healthy reaction. The patient was emaciated and feverish, his limbs tremulous, and face pallid, although these symptoms might be owing in some measure to his recent dissipation.

Notwithstanding that his pulse was full and regular, he was at once put upon generous diet and sustaining treatment, taking twenty-five drops of the tincture of the chloride of iron three times a day; while the nitric acid lotion, in the strength of five drops of the acid to an ounce of mucilage of gum-arabic was employed locally. The inflamed edges were freely painted with the tincture of iodine, diluted with an equal quantity of alcohol, and the granulations, as soon as they appeared, were dressed with simple cerate upon patent lint. The whole organ was enveloped in an emollient poultice, and kept at rest and elevated.

The beneficial effects of this course were manifested almost immediately. The pain disappeared in a few hours; the margins of the ulcer assumed a healthy appearance, and the sloughs speedily separated. The patient soon recovered his strength, and before three weeks had passed cicatrization was rapidly taking place over the diseased surface. No morbid erections or unpleasant symptoms appeared at any time, nor were there any lymphatic enlargements or secondary consequences. A portion of the head of the penis, about as large as a marble, nearly all of the spongy portion, and the lower half of the cavernous bodies still remain; but all the prepuce and the greater part of the skin over the organ were lost. A little superficial sinus, running up subcutaneously toward the pubic symphysis, is still open, but is healing rapidly under the use of mild injections.

*Remarks.*—Perhaps the most favorable circumstance which can happen to a patient suffering with well-confirmed chancre, is a little sloughing; for in this process the specific virus is generally destroyed so that no constitutional symptoms follow. As in this case, the upper portion of the prepuce is usually the part first attacked, the gangrenous action sometimes merely perforating it, but often involving the remainder of the penis. There is at first an increase of feverish excitement, followed eventually, however, by typhoid symptoms, which constitute the real danger of this form of disease. In treating it we must watch closely the indications, using depressants or stimulants according to the state of the system. Locally, our object must be to arrest the gangrene and favor the detachment of the slough, making use of iodine and medicated poultices, with mild escharotics to the dying mass, relieving all constriction, allaying fetor and promoting cleanliness and rest. The reparative process is best advanced by the nitric acid lotion with laudanum or dilute citrine ointment, and warm water-dressings or soothing poultices.

*Fibroid Tumor of the Prepuce; Removed by Excision.*

Charles S., aged twenty-four, single, contracted syphilis several years since, but had been comparatively free from evil consequences until some months ago, when he had a number of preputial warts, for which he neglected to use any medical treatment. The result was a permanently œdematous and hardened condition of the retracted prepuce, which had steadily increased in size until the whole under side of the penis was occupied by an ovoidal tumor, nearly three inches in diameter. When first admitted, an indistinct fluctuation could be detected in its lower portion, and it had accordingly been treated by acupuncture and sorbefacient applications with the effect of slightly reducing and solidifying it; but of late it had been quite stationary. The penis had become thin and flabby and much incurvated, though there were occasional imperfect attempts at an erection.

The patient being very desirous of relief from the inconvenience and necessary impotence, the tumor was removed by excision, two cutaneous flaps being left, one on each side, which, after the operation, were approximated with silver-wire sutures. He made an excellent recovery, there being complete union throughout. Some hemorrhage attended the operation, but was readily controlled by ligatures.

Professor Gross remarked that this tumor was essentially an hypertrophy of the skin and cellular tissue, caused by the long-continued œdema of the prepuce, which was itself produced by the irritation of the venereal excrescences. The cellular structure in this situation is remarkably abundant, as well as extremely lax, and therefore readily admits of the infiltration of serum and even of plastic matter, especially if the morbid action be at all severe or protracted. In this case, the lymph, after organization, had undergone the fibrous degeneration, and though itself only incommoding by its bulk, might in time become the seat of more serious disease.

Interesting medico-legal questions may arise from a case like this, as to the competency of the patient to enter the married state. Encumbered and mutilated as the organ was, it would be impossible to declare upon oath that copulation or even actual impregnation could not take place.

*Internal Hemorrhoids.*

*Case* 1.—James McC., a laborer, unmarried, thirty-two years old, had suffered for years with internal piles. He was habitually costive, and could never empty his bowels without great pain, and not unfrequently loss of blood. He felt a constant sense of weight and oppression in the anal region, but, except this trouble, his general health was good.

The bowel having been forced to protrude over a vessel, the patient was placed upon his knees and elbows on the table, and the three tumors which were exposed seized, one by one, with a tenaculum, until a large, strong ligature could be firmly tied around the base of each. As they were of large size and had been disposed to bleed, the ends of the thread, instead of being cut off, were allowed to hang down beyond the anus, to assist in bringing down the bowel in case of secondary hemorrhage.

The ligatures and tumors came away between the fifth and ninth days; the bowels were then gently acted on by a mild laxative, and the rectum washed out every day with a full enema of cold water. The result was a perfect cure.

*Case* 2.—Daniel M., a shoemaker, aged twenty-nine, presented himself at the hospital, on account of several external hemorrhoids of medium size, which, at the time of his admission, were much inflamed and exceedingly tender and painful. The suffering was in great measure relieved by washing them every day with Castile soap and cold water, and by attention to his bowels; but a cure was finally effected by incising the tumors and turning out the dried and hardened contents.

*Remarks.*—In these two cases very different affections are presented under nearly the same name. An external pile, as it is commonly called, is simply an extravasation of blood, a sort of apoplectic clot, in the cellular tissue at the verge of the anus, caused by anything which provokes a rupture of a hemorrhoidal vein, followed by an escape of its contents. The blood coagulating, forms a hard lump, which, if proper care be not taken, becomes inflamed and excessively painful. Great as is the annoyance it produces, nothing is easier to cure, as the only essential indication is to get rid of the troublesome effusion by a free incision. Relief is thus afforded in a few seconds, and the patient is able to go about his business. But an internal hemorrhoid is a far more serious affection, being in reality an erectile tumor or aneurism by anastomosis, arising from a dilatation of the minute capillaries of the inferior hemorrhoidal arteries and veins. As they are covered only by the mucous membrane, they not unfrequently give rise to considerable loss of blood, and sometimes occasion the most distressing pain, especially in defecation. Horseback riding, a standing posture, habitual costiveness or diarrhœa, in short, anything which promotes local congestion, greatly aggravate their symptoms; yet it is remarkable that they often exist for years without seriously affecting the general health. Much may be done to relieve them by local means, as cooling enemata and mild astringent lotions, with emollient poultices, leeches, or ointments, if they become inflamed or protrude. Attention to the diet, an occasional aperient, and evacuating the bowels while in a recumbent posture, are also serviceable.

The only permanent benefit is derived from their removal, which should be effected with the ligature, the bowel having been protruded over a vessel, an act which is rendered much easier by immersing the hips for some time in warm water. If the tumor has a very broad base, a curved needle with a double thread may be passed through it so as to ligate it in two portions. Excision ought not to be thought of, and the écraseur is more painful, while the danger of secondary hemorrhage and stricture of the rectum is far greater than when use is made of the ligature.

*Hydrocele treated by Seton.*

*Case* 1.—Thomas W., aged fifty, was tapped three months previously, and twelve ounces of fluid drawn from the scrotum. The tumor soon reappeared, and at the time of the operation was quite large, of globular form, fluctuating distinctly, and rapidly increasing. Injection of tincture of iodine, diluted with six parts of alcohol, was first tried; but as this failed to secure the requisite amount of inflammation, a seton was introduced five days after the evacuation of the fluid, and allowed to remain four days, with the effect of completely obliterating the secreting cavity.

*Case* 2.—Charles G., the other patient, was a small, thin man, twenty-six years old, a tailor by trade, who had for ten years carried a large tumor in his scrotum, conical in form, which had never diminished in size or shown any material alteration. The testicle, as is occasionally the case, was found to be at the bottom of the swelling, which was elastic, but could hardly be said to fluctuate. The exploring needle indicated a large cavity with serous contents, and at once determined the diagnosis. Eleven ounces of thin, dark-colored coagulating fluid were drawn off; after which the trocar was reinserted in the canula and a counter opening made, through which was passed an eye-probe armed with a strip of muslin about a third of an inch in width, which was loosely tied in front of the scrotum. Forty hours afterward, the seton was withdrawn, as the swelling had in great measure reappeared, and its hardness indicated a sufficient effusion of lymph. A sorbefacient lotion of acetate of lead and opium was applied, and the scrotum kept suspended, until the end of the third week, when the cure was perfect.

*Remarks.*—Hydrocele may be confounded with hernia and sarcocele or disease of the testicle. Its irreducible character, elasticity, and the absence of impulse on coughing, as well as the history of its growth from below upward, its pyriform shape, the unaltered condition of the spermatic cord, and the usual position of the testicle at the junction of the inferior and middle third instead of at the bottom, are sufficient to distinguish it from the first of these affections; while its regular surface, smoothness, and translucency, with the fact of the peculiar sensitiveness of the testicle being still retained, separate it from the other. The only inconvenience it occasions arises from its bulk, and hence many practitioners content themselves with merely evacuating its serous contents as often as the fluid may accumulate; but if properly treated, the radical cure is at once easy and certain. The great desideratum is to procure an amount of inflammation sufficient to change the character of the serous membrane and secure obliteration of the sac, and the ordinary method of injecting such stimulating fluids as iodine, alcohol, port wine, lime-water, or astringent solutions is generally sufficient for this purpose. But the seton is far the most reliable mode, for when it is used we have complete control over the inflammatory action, and can carry it safely to the required point. A good rule is to wait until the tumor acquires about one-quarter of its original size, and has become well hardened from the effusion of lymph, before withdrawing the exciting cause of the irritation—a period generally of about forty-eight hours. In congenital hydrocele, where the cavity of the vaginal tunic connects with that of the peritoneum, it is usually sufficient to obliterate the canal by applying the pad of a truss over its course, and using sorbefacient lotions on the scrotum, which, of themselves, often effect a cure in children, and sometimes even in adults.

*Ununited Fracture of the Humerus, of Twenty-Seven Months' Standing, successfully treated by Excision and Silver Wire.*

Edward Shannon, aged thirty-two, of feeble constitution and dissipated habits, broke the humerus of his left arm about three inches above the elbow, twenty-seven months ago. He was under the influence of liquor at the time, and being away from home, neglected to have it thoroughly examined for more than three weeks after the accident. By that time there was great inflammation and suppuration, so that apparatus could not be properly applied, and in consequence there was no attempt at union, notwithstanding that pressure, friction of the broken ends, and even injection of iodine were tried. At the time of the operation there was great mobility at the seat of fracture, and though the patient had considerable use of his limb, it was nearly devoid of strength.

On the nineteenth of January, after chloroform had been administered, a straight incision, about four inches in length, was made through the posterior portion of the triceps muscle, exposing at once the membrane which had incased the fracture. On severing this, it was found that the oblique extremities had been rounded off and become incrusted by semi-cartilaginous tissue, so as to form a very perfect false joint, which was lubricated by an abundant thin, glairy fluid, though nothing like a synovial membrane could be detected. They were each in turn brought through the opening thus made, and cut off at right angles by means of a delicate saw introduced behind them. It was necessary to take about half an inch from the lower fragment and nearly three times as much from the other. One ligature, made of three strands of the usual sized silver wire, was passed horizontally through each section of bone, and another diagonally across the edges, which, after having been tightly twisted, were cut off short, and the ends bent up so as to lie close beside the body of the humerus. The incision through the skin was then approximated closely with interrupted sutures, and the whole arm placed in well-padded curved splints of binders' board.

For about forty-eight hours all went on well, but on the third day the patient began to feel considerable pain in the wounded arm, so that the dressings had to be removed, which it was hoped would not be necessary until a much later period. There was at this time considerable erysipelas, both in the city and hospital, and the edges of the incision, when the splints were taken off, were found to be affected with that disease, which rapidly became phlegmonous; the abscesses extending up over the shoulder and down below the elbow, the suppuration being very extensive. Supportive treatment was commenced at once, consisting of good diet, milk-punch, and the tincture of the chloride of iron; with local applications of dilute tincture of iodine and poultices of linseed meal. The patient's strength was reduced quite low, but he finally reacted; and when at last he was able to go out into the open air, he convalesced rapidly.

During this period, of course but little could be done, by apparatus or otherwise, to secure the fragments of the broken bone in the proper situation, and it was therefore quite unexpected to find that very decided union had taken place. The patient is now again wearing the splints, and there is every prospect of his having a strong and useful limb. Indeed, when last seen, on the twenty-third of May, nearly four months after the operation, the union was almost complete; a large quantity of callus had been thrown out, the elbow-joint had regained its motion, and the general health was excellent.

